# Supplementary material for: Conformational Changes and Unfolding of β-Amyloid Substrates in the Active Site of γ-Secretase
Source: Int J Mol Sci. 2024 Feb 22;25(5):2564. doi: 10.3390/ijms25052564 (PMC10932356; doi:10.3390/ijms25052564)
Supplement: Supplementary file 1 [file ijms-25-02564-s001.zip › ijms-2854183-supplementary.pdf]

Supplementary materials

Conformational changes and unfolding of β-amyloid substrates in the active site of γ-secretase

Jakub Jakowiecki, Urszula Orzeł, Przemysław Miszta, Krzysztof Młynarczyk, Sławomir Filipek

Contents

Figures S1–S4: Plots of force and work/energy for SMD simulations during unfolding of Aβ_{49}, Aβ_{46}, Aβ_{43}, and Aβ_{40} in GS, respectively.

Figures S5–S8: Timeline plots for SMD simulations during unfolding of Aβ_{49}, Aβ_{46}, Aβ_{43}, and Aβ_{40} in GS, respectively.

Tables S1–S4: Calculated work/energy and distances for SMD simulations during unfolding of Aβ_{49}, Aβ_{46}, Aβ_{43}, and Aβ_{40} in GS, respectively.

Table S5. Tokens to individual SMD simulations for GS-SMD server results page.

Figure S1. Plots of force (on left) and work/energy (on right) for eight SMD simulations (repeats) of unfolding of Aβ49. The vertical orange line indicates a frame with the closest distance of scissile bond to the catalytic residues (trimming conditions). Units: Force [pN], Work [kJ/mol], Time [ns].

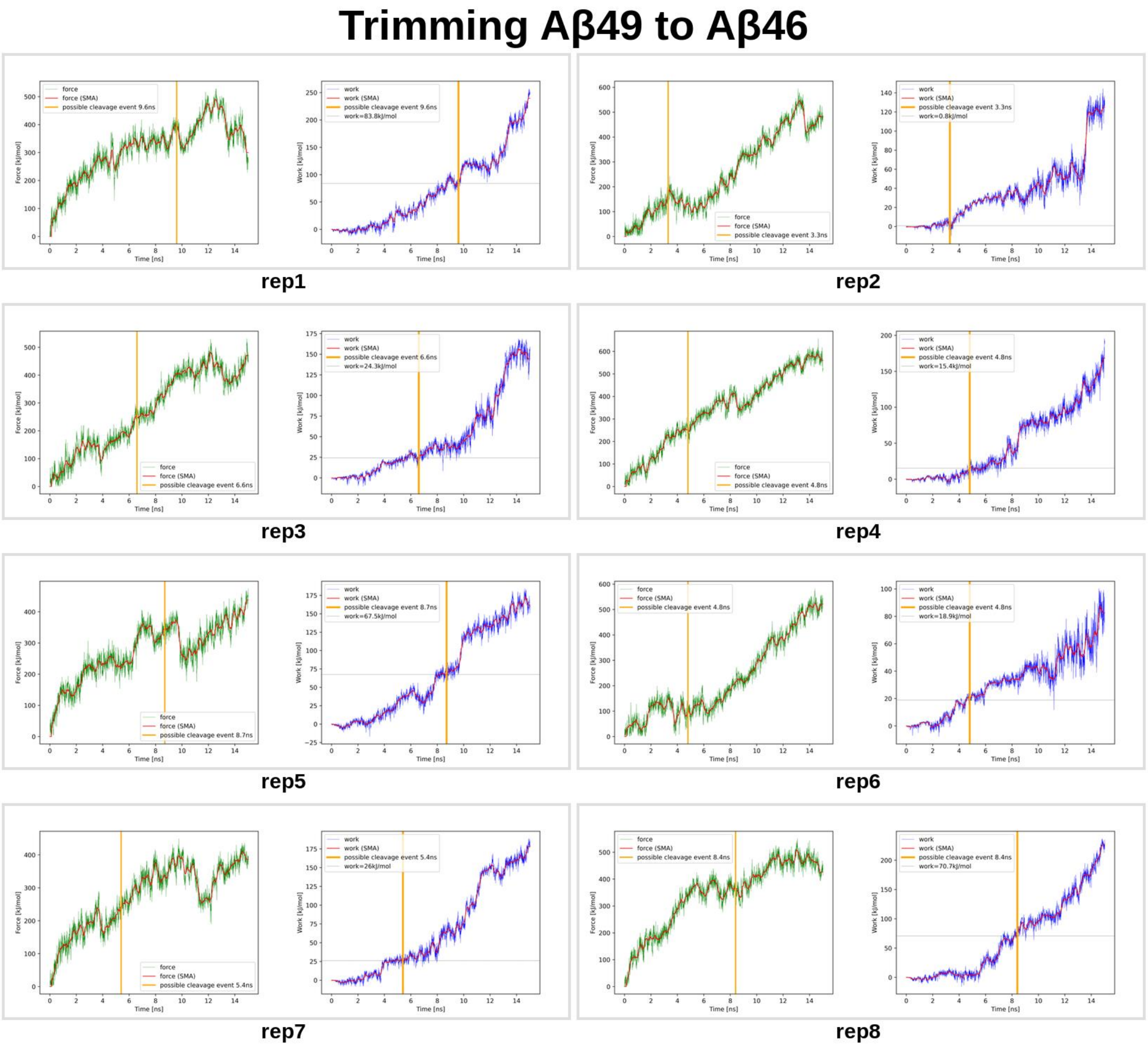

Figure S2. Plots of force (on left) and work/energy (on right) for eight SMD simulations (repeats) of unfolding of Aβ$_{46}$. The vertical orange line indicates a frame with the closest distance of scissile bond to the catalytic residues (trimming conditions). Units: Force [pN], Work [kJ/mol], Time [ns].

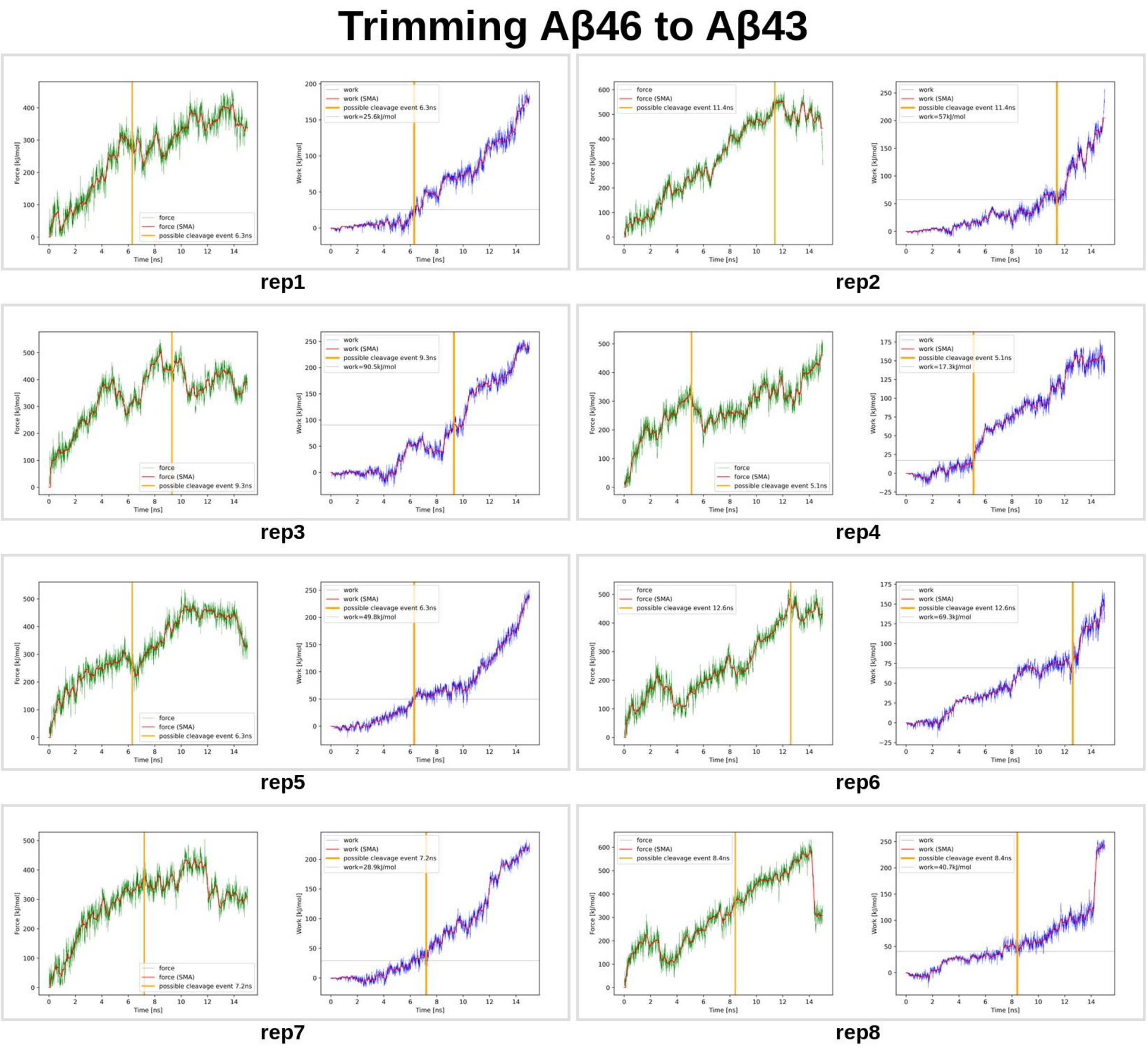

Figure S3. Plots of force (on left) and work/energy (on right) for eight SMD simulations (repeats) of unfolding of Aβ_{43}. The vertical orange line indicates a frame with the closest distance of scissile bond to the catalytic residues (trimming conditions). Units: Force [pN], Work [kJ/mol], Time [ns].

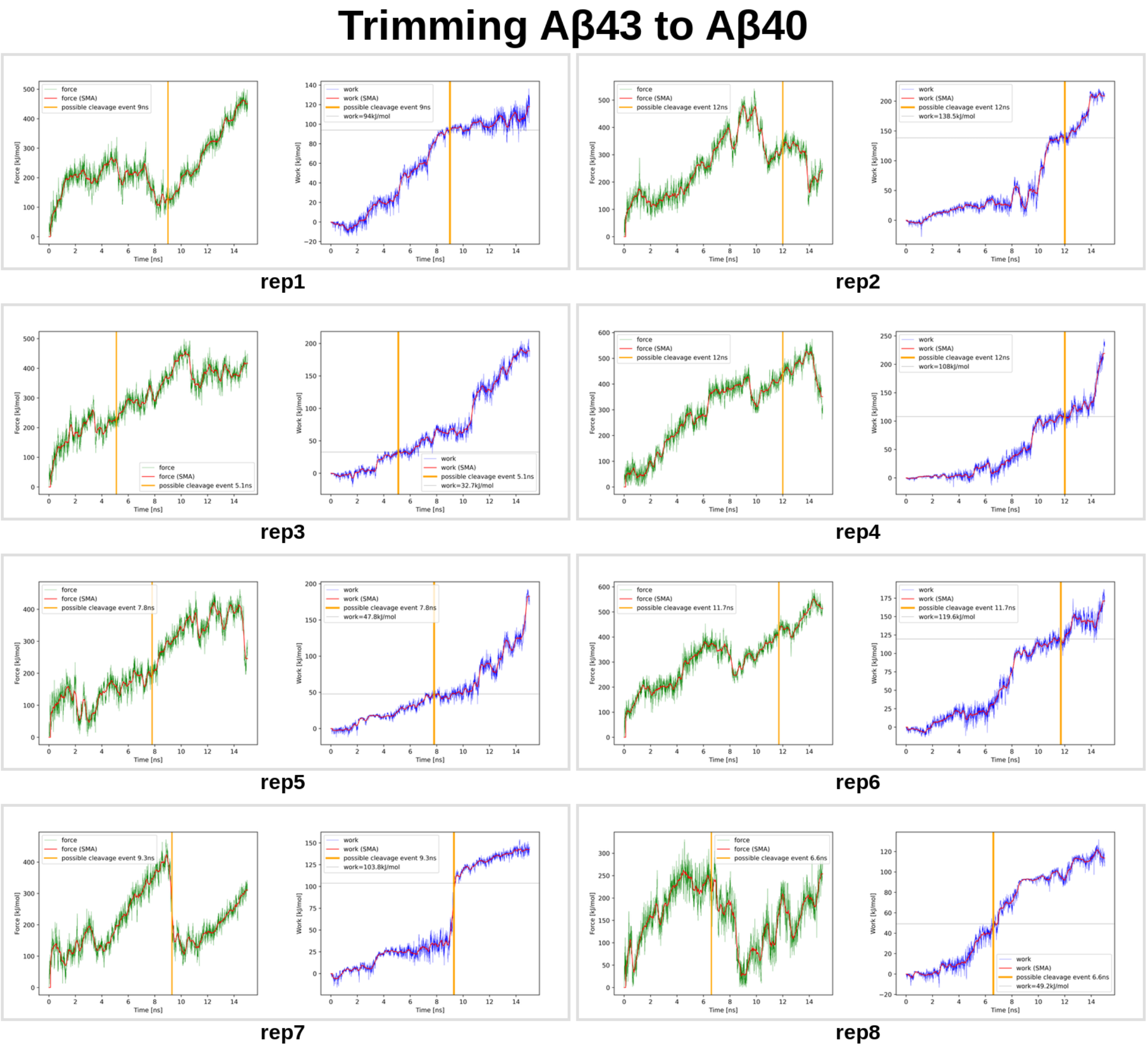

Figure S4. Plots of force (on left) and work/energy (on right) for eight SMD simulations (repeats) of unfolding of Aβ40. The vertical orange line indicates a frame with the closest distance of scissile bond to the catalytic residues (trimming conditions). Units: Force [pN], Work [kJ/mol], Time [ns].

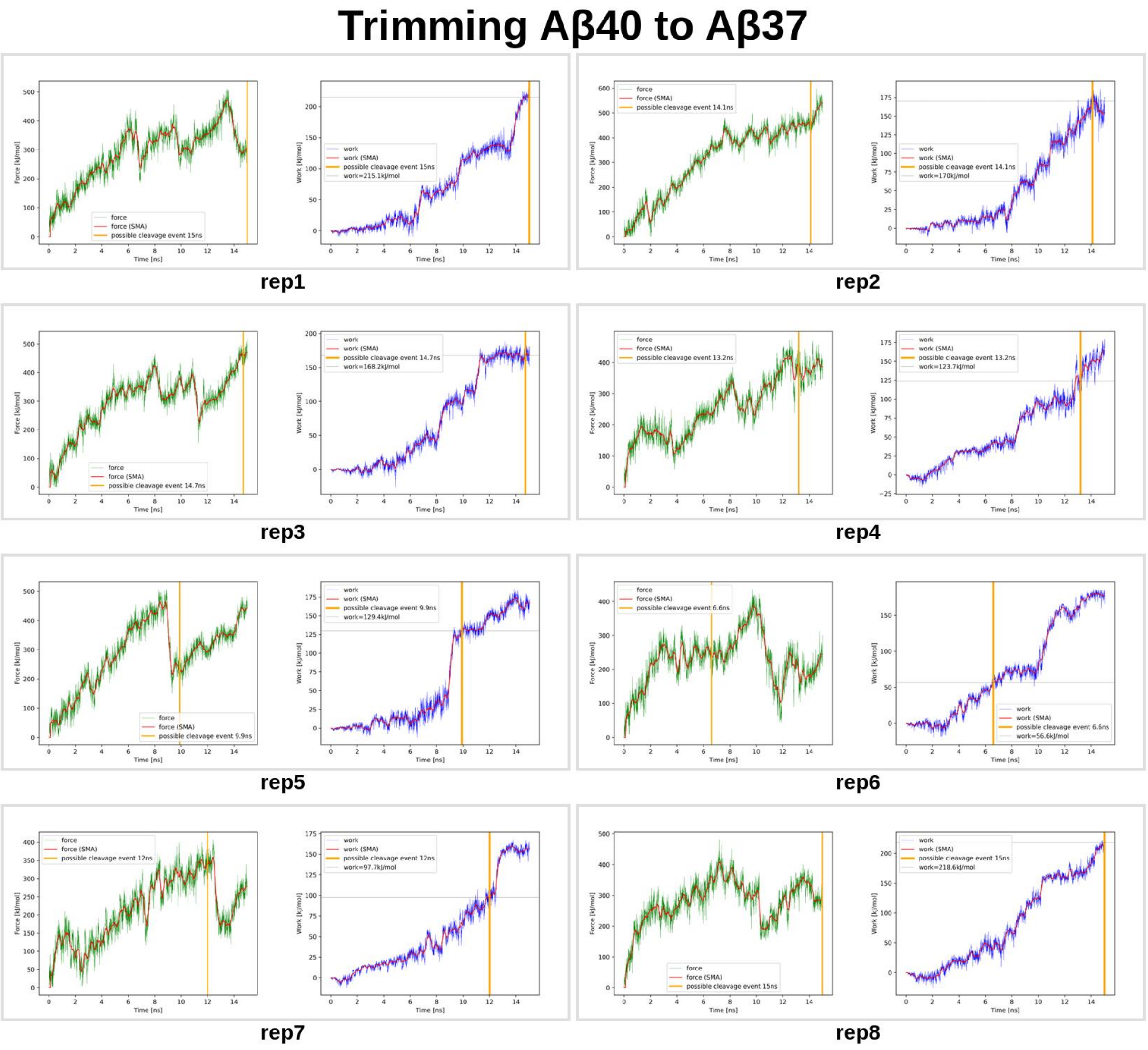

Figure S5. Timelines showing the secondary structure of Aβ49 during its unfolding. The last residue (33) is Leu49. The white window indicates a frame with the closest distance of scissile bond to the catalytic residues (trimming conditions).
Abbreviations: T – Turn, C – Coil, B – Bridge, E – Strand, I – π-Helix, G – 3_{10}-Helix, H – α-Helix.

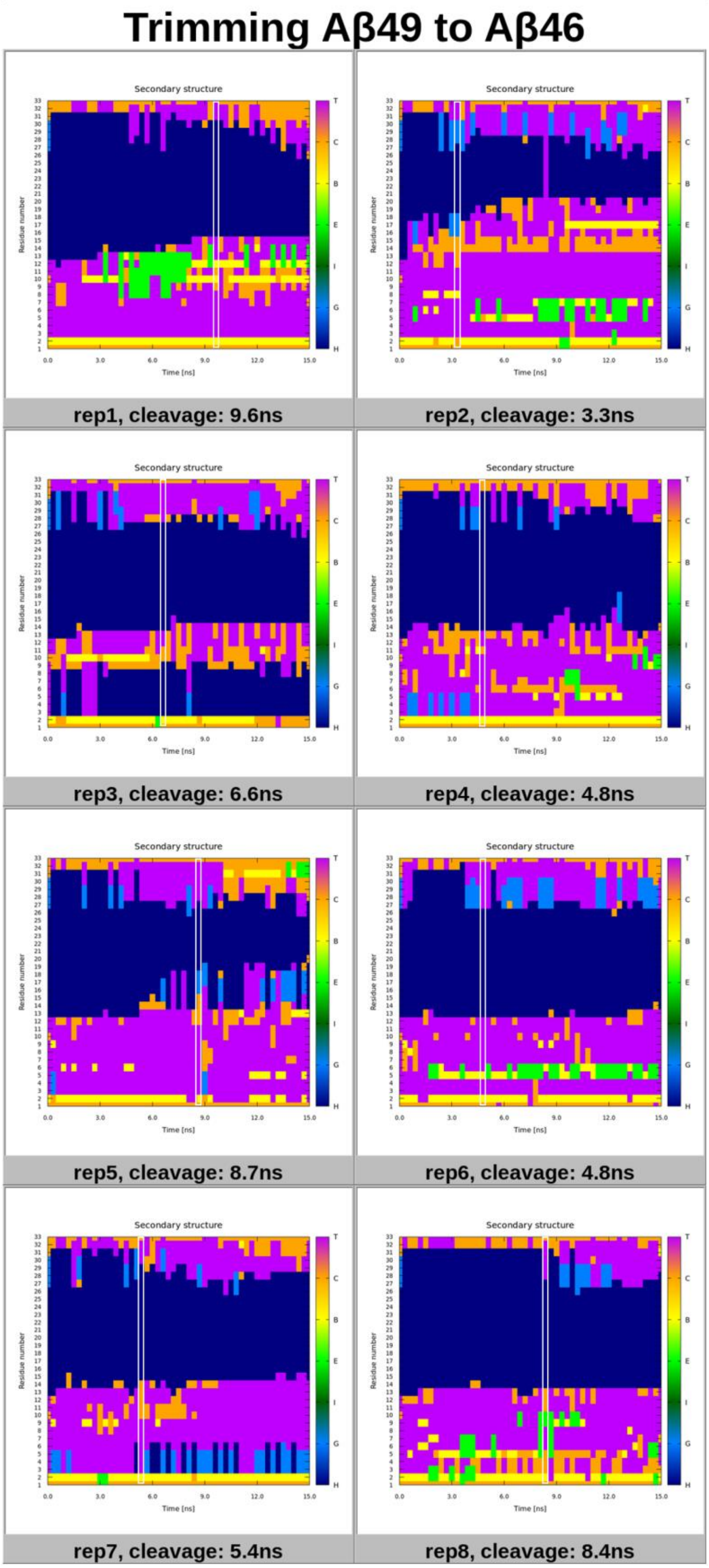

Figure S6. Timelines showing the secondary structure of $A\beta_{46}$ during its unfolding. The last residue (33) is Val46. The white window indicates a frame with the closest distance of scissile bond to the catalytic residues (trimming conditions).
Abbreviations: T – Turn, C – Coil, B – Bridge, E – Strand, I – π-Helix, G – 3_{10}-Helix, H – α-Helix.

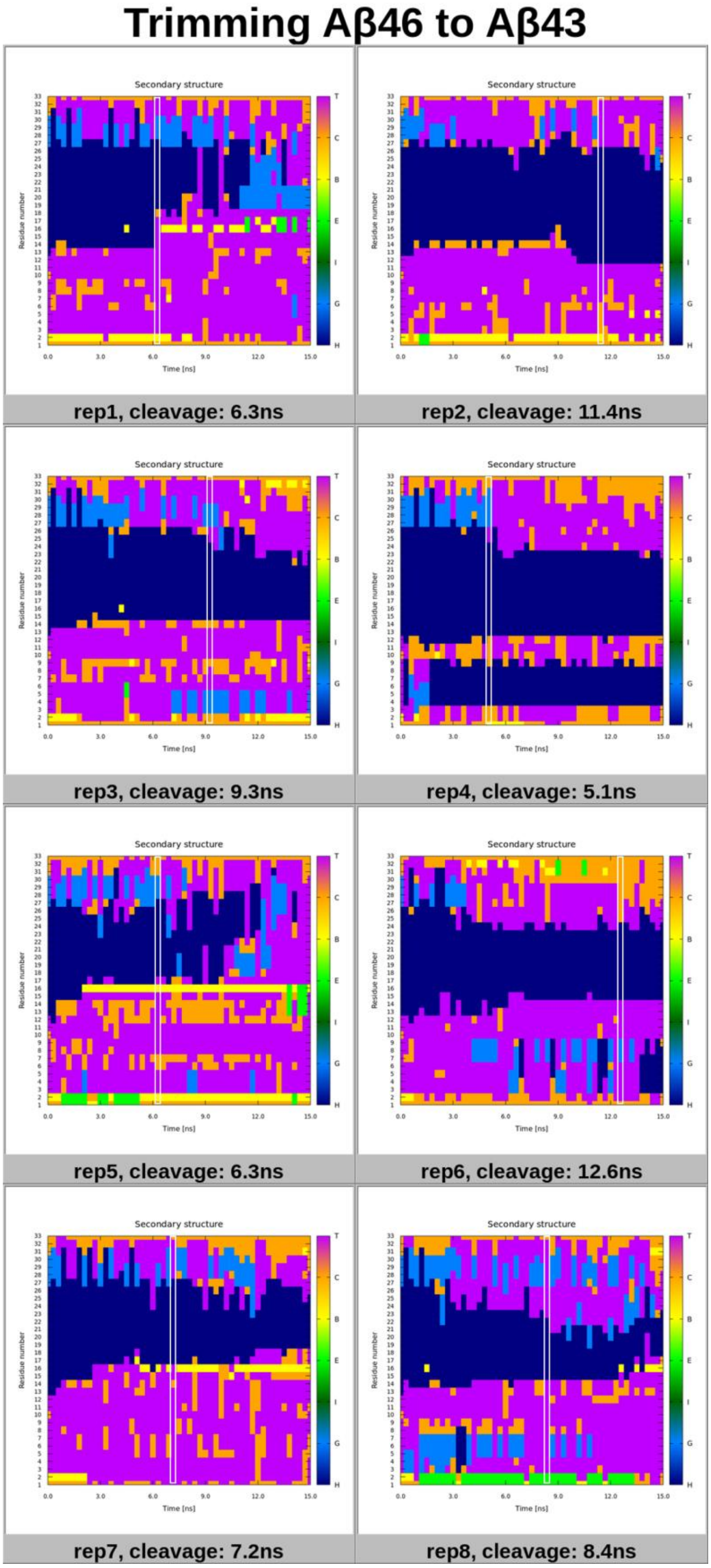

Figure S7. Timelines showing the secondary structure of Aβ43 during its unfolding. The last residue (33) is Thr43. The white window indicates a frame with the closest distance of scissile bond to the catalytic residues (trimming conditions).
Abbreviations: T – Turn, C – Coil, B – Bridge, E – Strand, I – π-Helix, G – 3_{10}-Helix, H – α-Helix.

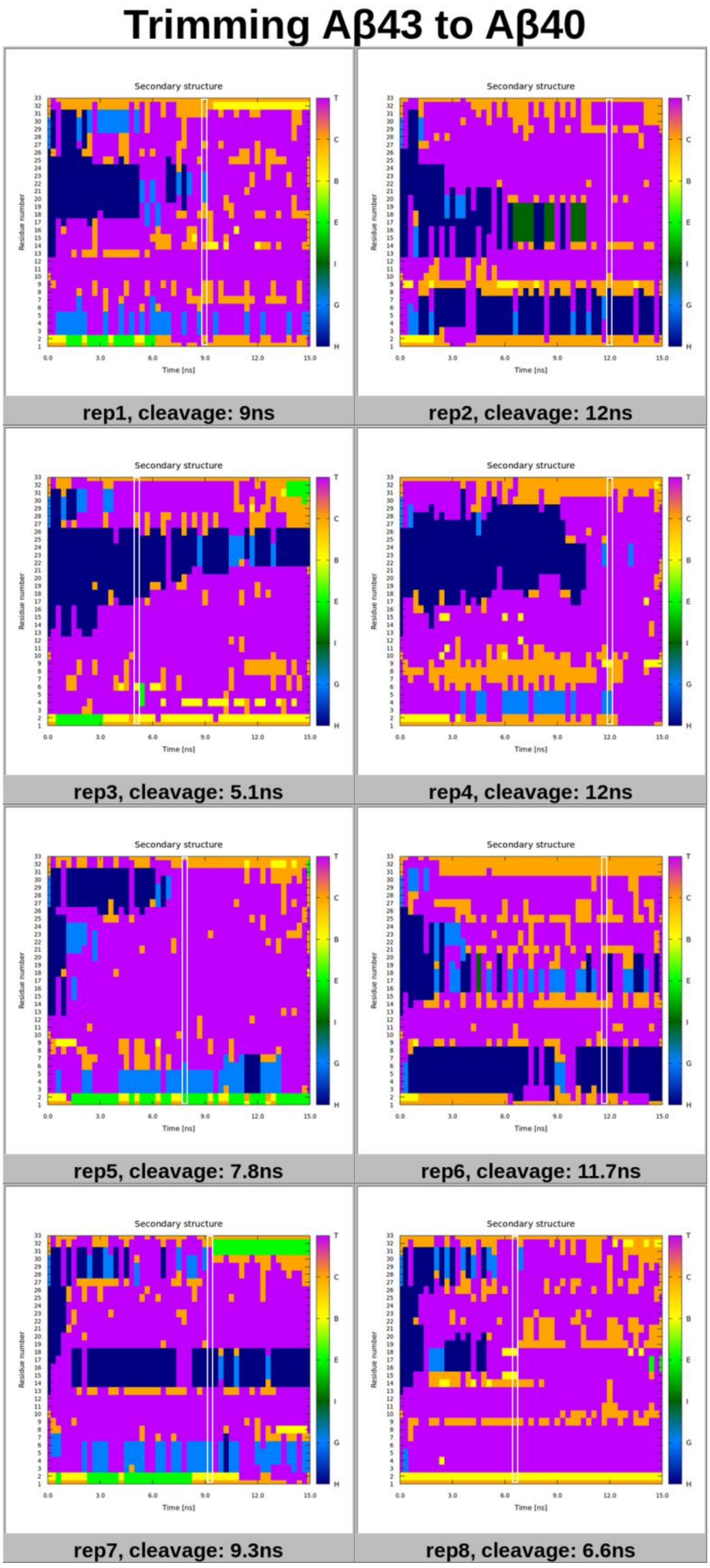

Figure S8. Timelines showing the secondary structure of Aβ$_{40}$ during its unfolding. The last residue (33) is Val40. The white window indicates a frame with the closest distance of scissile bond to the catalytic residues (trimming conditions).
Abbreviations: T – Turn, C – Coil, B – Bridge, E – Strand, I – π-Helix, G – 3$_{10}$-Helix, H – α-Helix.

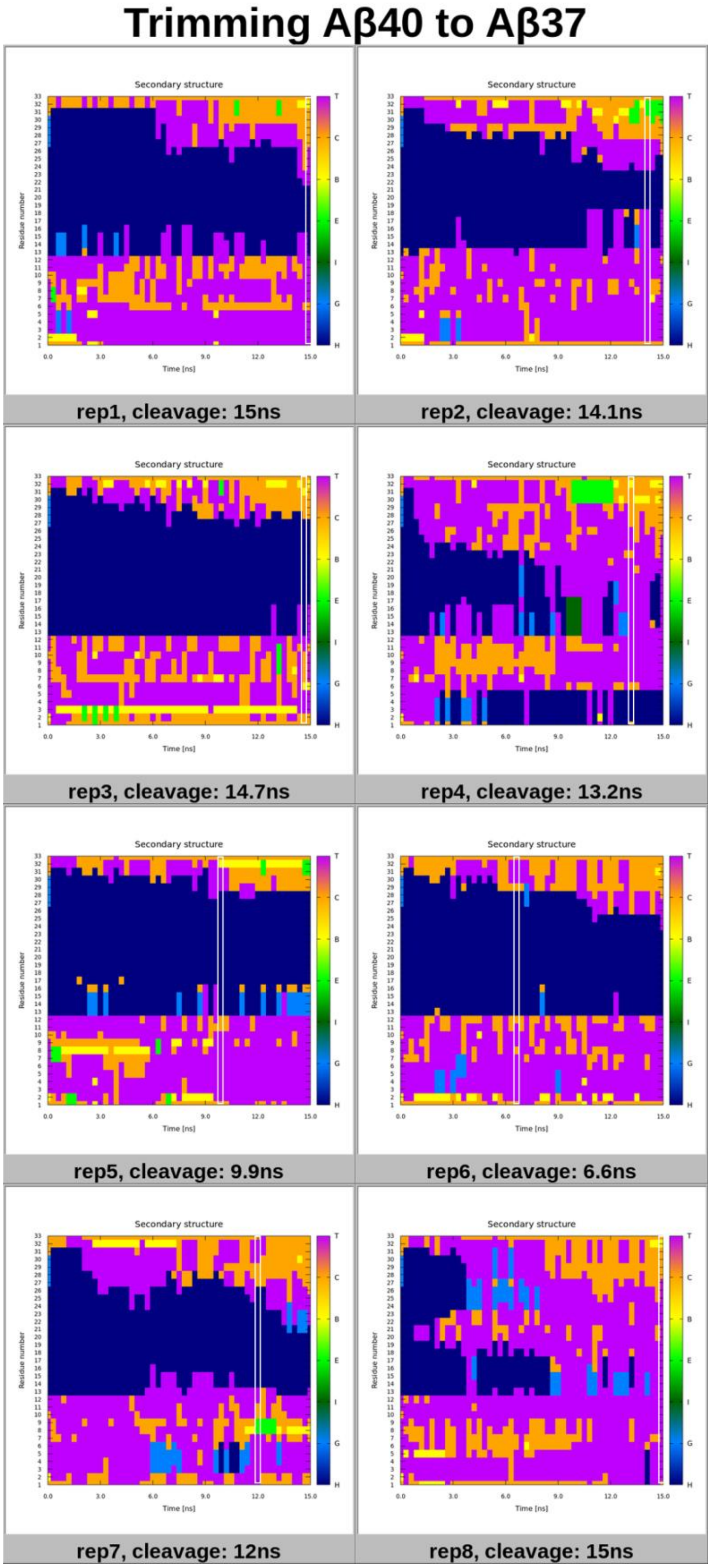

Table S1. Calculated work/energy and distances for SMD simulations of unfolding of Aβ_{49} in GS. For each simulation the work/energy is calculated for that frame with the shortest sum of distances of Val46 (n-3 residue, scissile bond) to the catalytic residues (Asp257 and Asp385). Distances are given in [Å].

**Trimming 1. Aβ_{49}→Aβ_{46}**							
**Sim no.**	**Frame**	**Time [ns]**	**Work [kJ/mol]**	**Val46–Asp257**	**Val46–Asp385**	**Shortest sum of distances**	**Asp257–Asp385**
**1**	32	9.6	83.8	3.97	6.80	10.77	7.21
**2**	11	3.3	0.8	4.20	6.29	10.49	6.34
**3**	22	6.6	24.3	4.66	6.13	10.79	6.27
**4**	16	4.8	15.4	5.71	4.35	10.06	3.82
**5**	29	8.7	67.5	3.51	6.60	10.11	7.79
**6**	16	4.8	18.9	5.19	4.09	9.28	5.70
**7**	18	5.4	26.0	4.80	3.87	8.67	5.99
**8**	28	8.4	70.7	4.87	4.79	9.66	6.21
		**Mean:**	**38.4**	**4.6**	**5.4**	**10.0**	**6.2**
		**STDEV:**	**30.8**				

Table S2. Calculated work/energy and distances for SMD simulations of unfolding of Aβ$_{46}$ in GS. For each simulation the work/energy is calculated for that frame with the shortest sum of distances of Thr43 (n-3 residue, scissile bond) to the catalytic residues (Asp257 and Asp385). Distances are given in [Å].

**Trimming 2. Aβ$_{46}$→Aβ$_{43}$**							
**Sim no.**	**Frame**	**Time [ns]**	**Work [kJ/mol]**	**Thr43–Asp257**	**Thr43–Asp385**	**Shortest sum of distances**	**Asp257–Asp385**
**1**	21	6.3	25.6	3.74	4.81	8.55	5.73
**2**	38	11.4	57.0	4.09	4.43	8.52	6.41
**3**	31	9.3	90.5	5.44	4.69	10.13	6.60
**4**	17	5.1	17.3	4.24	6.19	10.43	7.28
**5**	21	6.3	49.8	3.76	7.13	10.89	7.55
**6**	42	12.6	69.3	6.17	4.04	10.21	6.40
**7**	24	7.2	28.9	4.35	6.12	10.47	7.88
**8**	28	8.4	40.7	3.83	5.93	9.76	6.98
		**Mean:**	**47.4**	**4.5**	**5.4**	**9.9**	**6.9**
		**STDEV:**	**24.5**				

Table S3. Calculated work/energy and distances for SMD simulations of unfolding of Aβ_{43} in GS. For each simulation the work/energy is calculated for that frame with the shortest sum of distances of Val40 (n-3 residue, scissile bond) to the catalytic residues (Asp257 and Asp385). Distances are given in [Å].

Trimming 3. Aβ_{43}→Aβ_{40}							
**Sim no.**	**Frame**	**Time [ns]**	**Work [kJ/mol]**	**Val40–Asp257**	**Val40–Asp385**	**Shortest sum of distances**	**Asp257–Asp385**
**1**	30	9	94.0	6.41	4.32	10.73	4.47
**2**	40	12	138.5	6.13	3.78	9.91	5.04
**3**	17	5.1	32.7	3.83	4.67	9.34	6.15
**4**	40	12	108.0	3.02	7.15	10.17	7.50
**5**	26	7.8	47.8	4.66	4.73	9.39	6.44
**6**	39	11.7	119.6	3.79	3.74	7.53	5.25
**7**	31	9.3	103.8	5.64	5.18	10.82	6.96
**8**	22	6.6	49.2	3.50	6.88	10.38	7.49
		**Mean:**	**86.7**	**4.6**	**5.1**	**9.8**	**6.2**
		**STDEV:**	**38.5**				

Table S4. Calculated work/energy and distances for SMD simulations of unfolding of Aβ_{40} in GS. For each simulation the work/energy is calculated for that frame with the shortest sum of distances of Gly37 (n-3 residue, scissile bond) to the catalytic residues (Asp257 and Asp385). Distances are given in [Å].

Trimming 4. Aβ40→Aβ37							
**Sim no.**	**Frame**	**Time [ns]**	**Work [kJ/mol]**	**Gly37–Asp257**	**Gly37–Asp385**	**Shortest sum of distances**	**Asp257–Asp385**
1	50	15	215.1	3.09	4.83	7.92	6.46
2	47	14.1	170.0	4.35	4.32	8.67	6.96
3	49	14.7	168.2	4.48	5.11	9.59	6.82
4	44	13.2	123.7	4.61	5.87	10.48	7.01
5	33	9.9	129.4	4.86	5.24	10.10	7.58
6	22	6.6	56.6	4.20	4.92	9.12	5.97
7	40	12	97.7	5.84	4.83	10.67	7.96
8	50	15	218.6	4.05	3.46	7.51	5.61
		**Mean:**	**147.4**	**4.4**	**4.8**	**9.3**	**6.8**
		**STDEV:**	**56.3**				

Table S5. Tokens to individual SMD simulations for GS-SMD server results page (https://gs-smd.biomodellab.eu/results/). The simulations can be viewed individually or be used for comparisons one on one or in groups on the GS-SMD server.

Job name	Token
**Aβ_{40}**	
AB40_GVV_rep1	95ef7a8f-b951-411e-97cc-a5c3a29045fa
AB40_GVV_rep2	f40bb875-a98e-41db-8b88-bfa29544528d
AB40_GVV_rep3	ab67d771-f283-4cd2-b73d-20a83e2173b7
AB40_GVV_rep4	6afb5591-d9f9-48bc-a030-6940c64390ea
AB40_GVV_rep5	5e9ec51c-d099-4764-90ac-f27a641ad947
AB40_GVV_rep6	4ef9830a-b3fb-4d86-82a5-703a352d7f12
AB40_GVV_rep7	a39292af-824a-4d6d-bae2-46bde5fd9a2b
AB40_GVV_rep8	5a503f70-6fb1-4251-b4e2-4ace4d238ffe
**Aβ_{43}**	
AB43_IAT_rep1	ecfb60a4-660c-41b7-827b-0cc6eee7b290
AB43_IAT_rep2	da73890b-5c80-4b3c-8d98-a667e8127c9f
AB43_IAT_rep3	00d84a03-2b67-485f-9c6e-52c99f2d79fc
AB43_IAT_rep4	c8cb08dd-e599-43d0-8a10-f01e66e9957b
AB43_IAT_rep5	afc863d4-370a-447f-a02f-b1e6c25a0d46
AB43_IAT_rep6	29e712aa-78c8-4653-9bda-457a8fa934f4
AB43_IAT_rep7	6033e75a-b41c-4195-94f4-2b3d668904cf
AB43_IAT_rep8	d3585d02-f0b4-467f-b9c4-6afbb484c450
**Aβ_{46}**	
AB46_VIV_rep1	6b0e09b8-50e4-4f89-9576-7d3a21121b42
AB46_VIV_rep2	f59acd4d-b5cc-49cb-9e13-ebb83e7bd094
AB46_VIV_rep3	220ce7db-e0a3-4a4b-aced-c6f2f8feeca2
AB46_VIV_rep4	7b295ea7-87dc-4eec-b233-6c3af362779d
AB46_VIV_rep5	eeabb493-56e1-434c-b700-0f5d86371398
AB46_VIV_rep6	b30239fb-db29-48b7-a572-1c1413af8e71
AB46_VIV_rep7	cb98a507-cfc1-424d-967f-fb63b6739337
AB46_VIV_rep8	8d1685e4-0071-47ff-be46-fff1f6df23d1
**Aβ_{49}**	
AB49_ITL_rep1	a92905ef-e173-463d-b5c6-a3707fab83f4
AB49_ITL_rep2	833b87a6-4233-487e-8000-3ed19f2b5fd9
AB49_ITL_rep3	a2709677-1148-4353-bd09-5ce171f74902
AB49_ITL_rep4	707f4729-8f33-483e-a42e-8f0f78303dc3
AB49_ITL_rep5	4f82cf20-dc22-4a4d-bfa7-fb184141432b
AB49_ITL_rep6	af47c4bf-9112-44d3-aede-fb2779d49d34
AB49_ITL_rep7	f4ea2889-911a-4592-b597-15763d7939c5
AB49_ITL_rep8	1e7f0b23-ab59-4f77-ac08-d1947c0711b1
